# Serum Neuropeptide Y: A Potential Prognostic Marker of Intracerebral Hemorrhage

**DOI:** 10.1155/2021/7957013

**Published:** 2021-08-10

**Authors:** Weiming Sun, Zhenxing Zhang, Xu Feng, Xin Sui, Ye Miao

**Affiliations:** ^1^Department of Breast Surgery, The First Affiliated Hospital of Jinzhou Medical University, Jinzhou, 121000 Liaoning Province, China; ^2^Department of Neurosurgery, The First Affiliated Hospital of Jinzhou Medical University, Jinzhou, 121000 Liaoning Province, China

## Abstract

**Objective:**

Neuropeptide Y (NPY), a 36-amino acid neuromodulator, is mainly secreted by neurons in the central and peripheral nervous systems, which participate in the regulation of a series of physiological processes. However, there are few studies on its correlation with intracranial hemorrhage (ICH). The purpose of this study is to determine whether the serum NPY level is related to the prognosis of ICH.

**Methods:**

364 patients diagnosed with ICH were included in the current study. The demographics, anthropometrics, medical history, clinical severity, and laboratory data are collected. Enzyme-linked immunoassay (ELISA) was used to detect the serum NPY level of each patient upon admission. Three months after the occurrence of ICH, we used the modified Rankin scale (mRS) to evaluate the prognosis of patients, and mRS > 2 was defined as a poor prognosis.

**Results:**

A total of 364 patients with ICH were included in the study, including 140 patients with a good prognosis and 224 patients with a poor prognosis. Compared with patients with a poor prognosis, ICH patients with a good prognosis have a lower baseline National Institutes of Health Stroke Scale (NIHSS) score (*p* = 0.036) and smaller hematoma volume (*p* = 0.039). The results of ELISA showed that compared with patients with a poor prognosis, ICH patients with a good prognosis had lower serum NPY levels (19.4 ± 3.7 vs. 27.6 ± 3.3 ng/ml, *p* < 0.001). Linear correlation analysis showed that the serum NPY level of ICH patients was significantly positively correlated with the baseline NIHSS score (*r* = 0.413, *p* = 0.041) and hematoma volume (*r* = 0.308, *p* = 0.026). Receiver operating characteristic (ROC) curve analysis showed that the sensitivity of the serum NPY level to predict the prognosis of ICH was 70.9%, the specificity was 72.6%, and the cut-off value was 24.2 ng/ml.

**Conclusions:**

The serum NPY level may be used as a predictor of ICH prognosis.

## 1. Introduction

Intracranial hemorrhage (ICH) refers to any bleeding within the skull, which destroys neurons or compresses the surrounding brain tissue and causes neurological dysfunction [1]. The global incidence of stroke ranges from 339 to 1,184 per 100,000, and the epidemiology of hemorrhagic stroke shows that the incidence is 232-270 per 100,000 [2, 3]. ICH accounts for 10-20% of all strokes and is an important cause of death and disability in an aging society [4]. The main cause of spontaneous ICH is hypertension but also includes cerebral arteriosclerosis, cerebral amyloid angiopathy, aneurysm, venous sinus thrombosis, and arteriovenous malformations [5]. The 2016 Global Burden of Disease report pointed out that ICH caused approximately 62.8 million disability-adjusted life-year (DALY) losses, which has become a compelling public health issue [3]. So far, no single treatment has been proven to improve the prognosis of ICH.

A neuropeptide is a small protein substance produced by neurons, which can exert a variety of physiological and behavioral processes by acting on different substrates [6]. Neuropeptide Y (NPY) was first discovered by Swedish scientist Tatemoto and his colleagues in 1982 [7]. NPY consists of 36 amino acids, and its gene is located at 7p15.1 in humans, including 3 introns and 4 exons [8, 9]. The structure of NPY is similar to the known peptide YY and pancreatic polypeptide. In the central nervous system, NPY is mainly distributed in the basal ganglia, amygdala, and nucleus accumbens of the human body, and it is also widely distributed in the cerebral cortex [10]. Neuropeptide Y molecules participate in certain signal transduction pathways through G protein-coupled receptors, thereby exerting a variety of physiological functions [11].

Studies have shown that NPY plays an important role in stress response and antianxiety properties [12]. Neuropeptide Y plays an important role in many physiological functions such as energy homeostasis, cognition, and food intake. In addition, NPY is also involved in circadian rhythm, energy metabolism, cognition, food intake, synaptic excitability, and cardiovascular function [13]. However, there are few studies on the relationship between NPY and ICH. The purpose of this study is to explore the correlation between NPY and the prognosis of ICH and to determine whether NPY can be used as a target for the prognosis of ICH.

## 2. Methods

### 2.1. Population

We included 364 ICH patients in the First Affiliated Hospital of Jinzhou Medical College from April 1, 2018, to March 31, 2020. The selection criteria for ICH patients are within 24 hours of the onset of ICH and confirmation by computed tomography (CT) as ICH. The exclusion criteria for ICH patients are as follows: mRS > 2 before admission; combination with trauma, aneurysm, and arteriovenous malformations; a history of tumor; ICH patients requiring surgical treatment; and multiple organ dysfunction such as the heart, liver, and kidney. Our research was approved by the Jinzhou Medical Ethics Committee, and all participants or family members authorized us to use these data for clinical research.

### 2.2. Baseline Data Collection

Baseline data of ICH patients were collected after enrollment. These data include demographics or anthropometrics, medical history, and clinical severity. Among them, demographics or anthropometrics include age, gender, and BMI. Medical history mainly includes whether there is a history of hypertension, diabetes, hyperlipidemia, and coronary heart disease. Evaluation indicators of clinical severity include NIHSS scores and hematoma volume. All baseline data are collected, recorded, and analyzed by dedicated personnel.

### 2.3. Neurological Assessment

The National Institutes of Health Stroke Scale (NIHSS) is designed to assess the neurological outcome and recovery of stroke patients. The main assessment content of NIHSS includes the level of consciousness, facial muscle function, limb strength, sensory function, ataxia, lateral neglect, extraocular movement, visual field, aphasia, and dysarthria. The NIHSS score ranges from 0 to 42, and the higher the score, the more serious the neurological deficit [14].

### 2.4. Imaging Analysis

CT is a quick and effective detection tool to confirm the diagnosis of ICH. After admission, all patients underwent head CT examination to confirm the diagnosis. Commercial Analyze Direct 11.0 software (Analyze Direct, Overland Park, MS, USA) was used to determine hematoma volume in CT images. The image analysis is performed by experienced imaging doctors, who are blind to the research object and research plan.

### 2.5. Laboratory Data

The venous blood of all ICH patients was collected by nurses within 24 hours of admission. The collected venous blood was allowed to stand at room temperature for 20 minutes and then centrifuged at high speed to obtain serum. Purchased ELISA reagents were used to detect serum NPY levels. A purchased ELISA reagent (RayBiotech, Inc. Georgia, USA) was used to detect serum NPY levels. The operation of ELISA refers to the product specification and previous research reports [15]. Other laboratory data (total cholesterol, triglycerides, LDL cholesterol, HDL cholesterol, and blood glucose) are tested using standardized laboratory techniques.

### 2.6. Prognostic Evaluation

After 3 months of ICH, the modified Rankin scale (mRS) was used to evaluate the prognosis for all patients. mRS was created by Scottish doctors in 1957 and now is a globally used tool to evaluate the degree of neurological disability. mRS is a 6-point neuroevaluation scale, with a score ranging from 0 to 5 [16]. It is generally believed that an mRS score > 2 is classified as a poor prognosis, and an mRS score ≤ 2 is classified as a good prognosis.

### 2.7. Statistical Analysis

Continuous variables are represented by the mean ± standard deviation, and the comparison between variables is performed by a *t*-test. Categorical variables are expressed by the number (%), and the comparison between the variables uses the chi-squared test. In order to clarify the correlation between serum NPY levels in ICH patients and baseline data, Pearson correlation was used for analysis. Receiver operating characteristic (ROC) curve analysis was used to evaluate the accuracy of serum NPY levels in predicting the prognosis of ICH. A *p* value of less than 0.05 is considered statistically significant. In this study, SPSS 20.0 (SPSS Inc., Chicago, IL, USA) was used for statistical analysis.

## 3. Results

### 3.1. Baseline Data

During the participant recruitment period, a total of 364 ICH patients were enrolled. According to the evaluation results of the mRS score after 3 months, we divided the participants into a good prognosis group (mRS ≤ 2) and a poor prognosis group (mRS > 2).  Table 1 summarizes the baseline data on the 3-month prognosis of all participants.

We collected demographics or anthropometrics (age, gender, and BMI), medical history (hypertension, diabetes, hyperlipidemia, and coronary heart disease), and laboratory data (total cholesterol, triglycerides, LDL cholesterol, HDL cholesterol, and blood glucose) of the two groups. The results showed that the difference between the above indicators between the poor prognosis group and the good prognosis group was not statistically significant (*p* > 0.05).

**Figure 1:**
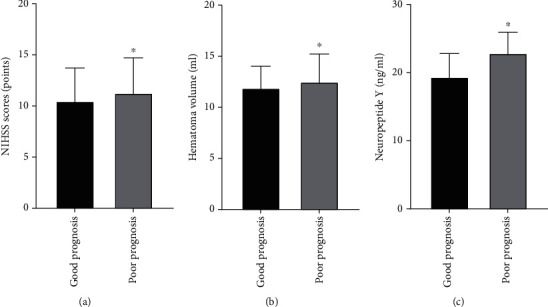
NIHSS scores, hematoma volume, and serum neuropeptide Y levels according to prognosis in patients with ICH: (a) NIHSS scores; (b) hematoma volume; (c) neuropeptide Y. NIHSS: National Institutes of Health Stroke Scale. Compared to the good prognosis group, ^∗^*p* < 0.05. Serum NPY level determination.

The clinical severity was evaluated by NIHSS scores and hematoma volume, which reflected the degree of neurological impairment in patients with ICH. The NIHSS scores of ICH patients in the good prognosis group and poor prognosis group were 10.5 ± 3.4 points and 11.3 ± 3.6 points, respectively. The hematoma volume of ICH patients in the good prognosis group and the poor prognosis group was 11.9 ± 2.3 ml and 12.5 ± 2.9 ml, respectively. As shown in Figure 1, the comparison of NIHSS scores (Figure 1(a)) and hematoma volume (Figure 1(b)) between the good prognosis group and the poor prognosis group of ICH patients was statistically significant (NIHSS scores, *p* = 0.036; hematoma volume, *p* = 0.039).

Peripheral blood NPY concentration results showed that the NPY levels in the good prognosis group and the poor prognosis group of ICH patients were 19.4 ± 3.7 ng/ml and 27.6 ± 3.3 ng/ml, respectively. As shown in Table 1 and Figure 1, the comparison of NPY serum levels between the two groups was statistically significant (*p* < 0.001).

### 3.2. Linear Correlation Analysis

The results of the correlation analysis are summarized in Table 2. The results of correlation analysis showed that serum NPY levels in ICH patients were not significantly correlated with age, BMI, total cholesterol, triglycerides, LDL cholesterol, HDL cholesterol, and blood sugar (*p* > 0.05). However, serum NPY levels in ICH patients were positively correlated with NIHSS scores (*r* = 0.413, *p* = 0.041). This positive correlation also appeared between the serum NPY level and hematoma volume (*r* = 0.308, *p* = 0.206).

### 3.3. ROC Analysis

**Figure 2:**
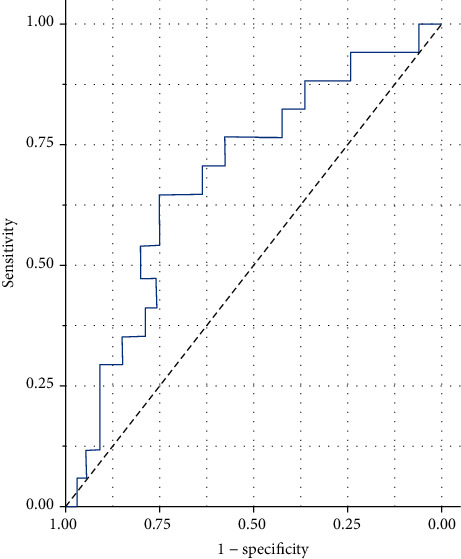
ROC analysis of serum NPY levels for the prognosis of ICH. ROC: receiver operating characteristic; NPY: neuropeptide Y; ICH: intracerebral hemorrhage.

In order to determine the diagnostic accuracy of serum NPY concentration on the prognosis of ICH, we adopted the method of ROC analysis. ROC analysis showed that the sensitivity of serum NPY concentration in diagnosing the prognosis of ICH was 70.9%, the specificity was 72.6%, and the cut-off value of NPY as a predictor was 24.2 ng/ml. The results of ROC analysis are shown in Figure 2.

## 4. Discussions

Our study compared the difference in serum NPY concentration between ICH patients with good prognosis and poor prognosis. Our study found that the serum NPY concentration of patients in the ICH prognosis group was significantly higher than that in the poor prognosis group. Through further research, we also found that serum NPY concentration was positively correlated with the NIHSS score and hematoma volume. These results suggest that serum NPY concentration may have potential value in predicting the prognosis of ICH. Therefore, we used ROC analysis to verify the accuracy of serum NPY in predicting the prognosis of ICH. Finally, we found that serum NPY can be used to predict the prognosis of ICH with high sensitivity and specificity, which can be used as a target for predicting the prognosis of ICH.

NPY is a neurotransmitter synthesized and released by neurons first identified from a pig brain. NPY is the most abundant peptide in the mammalian brain and is highly conserved throughout the evolutionary process [17]. NPY is highly homologous with YY peptide (PYY) and pancreatic polypeptide (PP), and their homology reaches 70% and 50%, respectively [18]. In recent years, NPY has been proven to play an important role in a variety of physiological conditions, such as vasoconstriction, ventricular remodeling, energy metabolism, food intake, and neuroendocrine axis regulation [19]. Therefore, identifying the specific receptors and related pathways of NPY is essential for understanding its specific molecular regulation and providing the possibility for the development of new therapeutic approaches.

NPY has been shown to be involved in the regulation of a variety of neurological diseases. Japanese scholars found in Osaka that the plasma NPY concentration of patients with Alzheimer's disease (AD) decreased, suggesting that NPY is involved in the pathogenesis of AD [20]. French scholars found that intracerebral administration of NPY can reduce the loss of dopamine (DA) and DA transporter in Parkinson's disease (PD) model mice, thereby reducing the symptoms of PD, suggesting that NPY has a certain therapeutic effect on PD [21]. Studies have also shown that NPY is involved in the pathogenesis of Huntington's disease (HD) [22]. The above studies all suggest that NPY plays an important function in neurodegenerative diseases. A recent Chinese study showed that the level of NPY in peripheral blood of epileptic children was significantly increased and was positively correlated with NIHSS score and Hamilton Anxiety (HAMA) scores, suggesting that NPY is involved in the onset of epilepsy [23]. A study by Peking University showed that plasma NPY is significantly reduced in Vasovagal Syncope, which may participate in the pathogenesis of Vasovagal Syncope by increasing total peripheral vascular resistance and reducing cardiac output [24]. A study in the United States showed that the increased number of monocytes infiltrated into the brain of NPY-deficient mice can aggravate the process of neurological diseases. NPY as a negative regulator of monocyte recruitment into the central nervous system provides a potential therapeutic target for inhibiting central nervous system infection [25]. Interestingly, the latest Chinese study found that the level of NPY in the spinal cord of elderly mice decreased, which may be one of the mechanisms of senile pruritus, suggesting that NPY may be a potential intervention target for alleviating pruritus symptoms in the elderly [26].

In recent years, the relationship between NPY and stroke has also been widely reported. The study by Yu et al. found that gene polymorphism in the NPY promoter region is an independent risk factor for ischemic stroke, indicating that NPY may be involved in the disease process of stroke [27]. Korean studies also confirmed that the polymorphism in the C-399T region of the NPY promoter may be an independent risk factor for ischemic stroke [28]. A Polish study found that the plasma NPY level of women with acute ischemic stroke decreased, suggesting that NPY may be involved in the pathological mechanism of acute ischemic stroke [29]. Studies have also pointed out that platelet NPY is an important neuroendocrine factor involved in ischemic vascular remodeling [30]. Schebesch et al. found in patients with subarachnoid hemorrhage (SAH) that the concentration of NPY in blood and cerebrospinal fluid increased, and it was also related to the degree of vasospasm, suggesting that NPY may be involved in the mechanism of vasospasm after SAH [31]. Although there are relatively many studies on NPY and SAH, the correlation between NPY and ICH prognosis has not been reported yet.

Our research has some limitations. First of all, we are a single-center study; secondly, we did not do dynamic detection of serum NPY levels; finally, we did not do long-term follow-up for the prognosis of ICH. Nevertheless, our study revealed for the first time the potential correlation between NPY and the prognosis of ICH.

## 5. Conclusions

Increased serum NPY levels may be a predictive target for the poor prognosis of ICH. The correlation between NPY and ICH is worthy of further study, which may provide a new path for the prevention and treatment of ICH.

## Figures and Tables

**Table 1:** Baseline characteristics of ICH patients according to different prognosis at 3 months.

Characteristics	3-month prognosis		*p* values
	Good (*n* = 140)	Poor (*n* = 224)	
Demographics or anthropometrics			
Age (years)	65.3 ± 7.9	65.8 ± 8.2	0.566
Gender, male/female	89/51	138/86	0.707
Body mass index (kg/m^2^)	25.4 ± 1.8	25.5 ± 1.6	0.581
Medical history			
Hypertension	81	135	0.649
Diabetes mellitus	30	52	0.692
Hyperlipidemia	76	118	0.765
Coronary artery disease	22	41	0.525
Clinical severity			
NIHSS scores (points)	10.5 ± 3.4	11.3 ± 3.6	0.036
Hematoma volume (ml)	11.9 ± 2.3	12.5 ± 2.9	0.039
Laboratory data			
Total cholesterol (mmol/l)	5.1 ± 1.1	5.0 ± 1.2	0.425
Triglycerides (mmol/l)	1.8 ± 0.3	1.8 ± 0.2	1.000
LDL cholesterol (mmol/l)	2.7 ± 0.6	2.8 ± 0.5	0.087
HDL cholesterol (mmol/l)	1.3 ± 0.3	1.3 ± 0.2	1.000
Blood glucose (mmol/l)	6.8 ± 0.9	6.9 ± 0.7	0.237
Neuropeptide Y (ng/ml)	19.4 ± 3.7	27.6 ± 3.3	<0.001

NIHSS: National Institutes of Health Stroke Scale; LDL: low-density lipoprotein; HDL: high-density lipoprotein.

**Table 2:** The association between serum neuropeptide Y levels and baseline characteristics.

Characteristics	*r*	*p* values
Age (years)	0.391	0.134
Body mass index (kg/m^2^)	0.237	0.252
NIHSS scores (points)	0.413	0.041
Hematoma volume (ml)	0.308	0.026
Total cholesterol (mmol/l)	0.265	0.218
Triglycerides (mmol/l)	0.174	0.347
LDL cholesterol (mmol/l)	0.206	0.279
HDL cholesterol (mmol/l)	-0.282	0.093
Blood glucose (mmol/l)	0.199	0.245

NIHSS: National Institutes of Health Stroke Scale; LDL: low-density lipoprotein; HDL: high-density lipoprotein.

## Data Availability

The data used to support the findings of this study are available from the corresponding author upon request.
